# Thrombospondin-1 Receptor CD47 Overexpression Contributes to P-Glycoprotein-Mediated Multidrug Resistance Against Doxorubicin in Thyroid Carcinoma FTC-133 Cells

**DOI:** 10.3389/fonc.2020.551228

**Published:** 2020-12-07

**Authors:** Marie-Pierre Courageot, Laurent Duca, Laurent Martiny, Emmanuelle Devarenne-Charpentier, Hamid Morjani, Hassan El Btaouri

**Affiliations:** ^1^Laboratoire de Biospectroscopie Translationnelle (BioSpecT), UFR Pharmacie, Université de Reims Champagne Ardenne, Reims, France; ^2^UMR-CNRS 7369 Matrice Extracellulaire et Dynamique Cellulaire (MEDyC), UFR Sciences Exactes et Naturelles, Université de Reims Champagne Ardenne, Moulin de la Housse, Reims, France

**Keywords:** chemo-resistance, doxorubicin, P-glycoprotein, TSP-1, CD47, thyroid carcinoma cells

## Abstract

It is now admitted that in addition to acquired resistance, the tumor microenvironment contributes to the development of chemo-resistance and malignant progression. In a previous study, we showed that Dox induced apoptosis in FTC-133 cells by trigging JNK pathway. This process was accompanied by a decrease of thrombospondin-1 (TSP-1) expression. Moreover, exogenous TSP-1 or its C-terminal-derived peptide interact with receptor CD47 and are able to protect FTC-133 cells against Dox-induced apoptosis. Here, we investigated the involvement of TSP-1/CD47 interaction in a context of acquired multidrug resistance in FTC-133 cells. To that end, we established a Dox-resistant cell line (FTC-133R cells) which developed a resistance against Dox-induced apoptosis. Cell viability was evaluated by Uptiblue assay, nuclear Dox was measured by microspectrofluorimetry, caspase activity was measured by fluorescence of cleaved caspase-3 substrate, gene expression was evaluated by RT-PCR and protein expression was examined by western-blot. Our results showed that FTC-133R overexpressed the P-gp and were 15-fold resistant to Dox. JNK phosphorylation and Dox-induced apoptosis were reduced in FTC-133R cells. Expression of CD47 was increased in FTC-133R cells but TSP-1 expression presented similar levels in two cell lines. VPL restored Dox nuclear uptake and FTC-133R cell sensitivity to apoptosis and induced a decrease in CD47 mRNA expression. Moreover, knockdown of CD47 in FTC-133R cells induced an increase in JNK activation and sensitized FTC-133R cells to Dox. Our data suggest that CD47 is able to contribute to the protection of FTC-133R cells against Dox-induced apoptosis and/or to potentiate the acquired Dox resistance.

## Introduction

Mechanisms associated with “acquired resistance” to cancer chemotherapy include decreased cellular incorporation of drugs, qualitative and/or quantitative modification of the therapeutic target, drug metabolism that decreases their effectiveness, repair of damage caused by the drug, and activation of anti-apoptotic pathways. In this case, some “predisposed” cells develop one of these mechanisms thus leading to the development of a “secondary resistant” tumor (1–3).

The main cause explaining the decrease of drug cellular incorporation is due to expression of several ATP Binding Cassette (ABC) transporters (4, 5). The first ABC transporter identified is the P-glycoprotein (P-gp) encoded by *ABCB1* gene (6, 7). The ABC proteins transport the anticancer drugs to the extracellular medium so leading to a decrease of drug concentration in the target cell nucleus. Such mechanism of resistance is called Multi-Drug Resistance (MDR). Several strategies have been developed to overcome this MDR, particularly by using small molecules able to inhibit ABC protein transport activity (8, 9). The first inhibitor described as able to inhibit P-gp and to restore sensitivity to anticancer drug is the Ca^2+^ channel inhibitor verapamil (VPL) (10–13).

However, the tumor cell escape from the drug cytotoxic effects can also involve a “*de novo* resistance”. Various factors present in the tumor cell microenvironment contribute to the development of this resistance (14, 15). On the one hand, interstitial proteins of the stroma, such as collagen and fibronectin, have been identified as adhesive factors able to induce resistance to chemotherapy by interacting with specific receptors and inducing survival signaling pathways (16–18). On the other hand, stromal soluble factors can also affect cancer cell survival. This is the case for TGFβ1 which sensitize ovarian carcinoma cells to paclitaxel (19). Thrombospondin-1 (TSP-1) is able to sensitize prostate carcinoma cells to the cytotoxic effect of taxol *via* its interaction with the CD47 receptor (20).

In previous works, we have reported that TSP-1 induced FTC-133 thyroid carcinoma cell survival and protection against apoptosis. In fact, camptothecin and doxorubicin (Dox), which inhibit topoisomerases I and II respectively, induced apoptosis in FTC-133 cells through the *de novo* synthesis of ceramides (21). We have showed that both drugs activated the c-Jun N-terminal kinase/Activating transcription factor-2 (JNK/ATF-2) pathway to induce apoptosis through a *de novo* synthesis of ceramide (22). This apoptosis was accompanied by a decrease of TSP-1 expression. Addition of exogenous TSP-1 protected cells against drug-induced apoptosis (23). Moreover, the anti-apoptotic role of TSP-1 involves its C-terminus part which interacts with the CD47 membrane receptor (23, 24).

In the present study, we have investigated how TSP-1/CD47 interaction can modulate the phenotype MDR. In order to perform this study, we established a Dox-resistant FTC-133 cell line (FTC-133R cell) by stepwise increasing drug concentration. We showed that FTC-133R cells are characterized by an overexpression of the P-gp and an increase of CD47 membrane receptor and develop a resistance to Dox-induced apoptosis by inhibiting Dox nuclear accumulation and preventing JNK pathway activation. The P-gp overexpression and TSP-1/CD47 interaction contributed to the development of this resistance. In fact, inhibition of P-gp function by VPL reduced CD47 and TSP-1 expression and sensitized FTC-133R cell to Dox-induced apoptosis by activating JNK pathway. Moreover, inhibition of CD47 expression by small interfering RNA (SiRNA) bypassed P-gp-induced resistance and restored the drug cytotoxicity by activating JNK pathway in FTC-133R cells. These data confirmed that the tumor microenvironment was a key player in the development of *de novo* chemoresistance, thereby influencing the development of acquired resistance. It is therefore possible to sensitize FTC-133R to chemotherapeutic treatment-induced apoptosis by acting directly on extracellular matrix components or by activating intracellular JNK pathway.

## Materials and Methods

### Materials

FTC-133 is a human follicular thyroid carcinoma derived cell line (ECACC94060901) obtained from a lymph node metastasis. Dox was obtained from Farmitalia (Italy). FTC-133R cells were selected from FTC-133 parental cells by stepwise increase of Dox concentration (from 10 to 400 nM) according to protocol of Chen et al. (25) modified. For the development FTC-133R, FTC-133 cells were incubated with 10 nM Dox and the drug concentration was doubled each time the treated cells reached the growth rate of the untreated cells, until the final concentration of 400 nM Dox was applied. Dulbecco’s modified Eagle’s medium/F-12, trypsin, and Lipofectamine RNAiMAX were purchased from Invitrogen (France). Foetal calf serum from Dutscher (France). TSP-1 monoclonal antibodies from ThermoFisher Scientific (France). Caspase-3, JNK, Phospho-JNK, CD47, and P-gp antibodies from Cell Signaling Technology (France). CD47 siRNA kit from Santa Cruz Biotechnology Inc (USA). ECL Western blotting detection reagents from Amersham (Germany). UptiBlue and BCA kit from Uptima (France). RNeasy^®^ Mini kit from Qiagen (France). CaspACE assay kit, AMV reverse transcriptase and oligo(dT) primers from Promega (France). Anisomycin, VPL, β-actin antibodies, and all other reagents from Sigma (USA).

### Cell Culture

FTC-133 and FTC-133R cells were cultured in 75 cm^2^ flasks at 37°C containing Dulbecco’s modified Eagle’s medium/F-12 (1:1) supplemented with 10% (v/v) heat inactivated foetal calf serum, 100 μg/ml streptomycin and 100 IU/ml penicillin in a 5% CO_2_/95% air-water saturated atmosphere [5]. After trypsinization, cells were cultured in 96-well plates for cell viability assay and in 6-well plates for flow cytometry, spectrofluorometry, western blot, mRNA extraction, cell transfection, and caspase assay.

### Cell Viability Determination

Cells were seeded into 96-well plates at a density of 10^4^ cells/ml for 24 h. The medium was then replaced with serum free medium with or without different concentrations of Mitoxantrone, camptothecin, anisomycin or Dox in presence or absence of VPL 1 µM. After 24 h incubation, 10% (v/v) UptiBlue was added during an additional 3 h. The viability was then measured by spectrofluorometry (λex: 530–560 nm; λem: 590 nm). Results were calculated as percent of control as follows: (experimental absorbance/untreated control absorbance) ×100.

### Nuclear Incorporation of Dox

Monitoring of the nuclear Dox incorporation was carried out using the microspectrofluorimeter M51 (Horiba Jobin Yvon France, Villeneuve d’Ascq). The cells were seeded in Petri dish 24 h prior to the measurements. After treatment with Dox 4 µM for 5 h, they were washed with PBS free of drug and placed in the medium without phenol red. To obtain fluorescence emission spectra, the 488 nm line was used with a ionized Argon laser (2065 series, SpectraPhysics, France). A nuclear spectrum of treated cells was obtained over the 500–700 nm wavelength range (26). The semi-quantification of nuclear Dox incorporation was obtained by measuring the fluorescence emission intensity of the band at 590 nm.

### Western Blot

After treatment, the cells were centrifuged (3,000 g, 5 min, 4°C) then washed with ice-cold PBS and lysed in an ice cold lysis buffer containing 10 mM Tris pH 7.4, 150 mM NaCl, 5 mM EDTA, 1 mM Na_3_VO_4_, 1 mM dithiothreitol, 10 μg/ml leupeptin, 10 μg/ml aprotinin, 10% (v/v) glycerol, 1% (v/v) Brij. The suspension was placed on ice for 20 min and then centrifuged (14,000 g, 15 min, 4°C). Total protein concentration was determined using BCA assay kit. Equal amounts of proteins were resolved by 10% (v/v) SDS-PAGE gels, transferred to a nitrocellulose membrane and then probed with the appropriate antibodies (monoclonal anti-TSP-1: dilution 1/400; polyclonal anti-caspase-3: dilution 1/1,000; polyclonal anti-P-gp: dilution 1/1,000; polyclonal anti-PhosphoJNK: dilution 1/1,000; polyclonal anti-JNK: dilution 1/1,000; polyclonal anti-CD47: dilution 1/1,000; monoclonal anti-β-actin: dilution 1/8,000). Horseradish peroxidase-conjugated goat anti-rabbit or anti-mouse IgG were used as secondary antibodies (dilution 1/4,000 and 1/10,000 respectively) and proteins were detected using an enhanced chemiluminescence kit.

### Caspase-3 Activity

After incubation, the cell were washed twice with PBS and scrapped with the ice-cold lysis buffer. Caspase-3 activity was measured by incubating 50 μg of cytosolic fraction with caspase-3 colorimetric substrate that absorbs at 405 nm following its cleavage. Absorbance was measured with a multichannel plate reader (Metertech. Inc. Σ960).

### Reverse Transcription-Polymerase Chain Reaction

RT-PCR was performed on total RNA prepared by RNeasy^®^ Mini kit (QIAGEN). One μg of total RNA was reverse transcribed using AMV reverse transcriptase and oligo(dT)15 primer. Amplification was performed using PCR Master Mix according the manufacturer’s instructions. The optimal reaction conditions were: 30 cycles, 56°C for TSP-1 and 25 cycles, 50°C for S26. Specific primer pairs were for CD-47: 5’-GATCAGCTCAGCTACTAT-3’ and 5’-ACAATGACAG TGATCACT-3’; for β-Actin: 5’-AGGCACCAGGGCGTGAT-3’ and 5’-GCCCACATAGGAATCCTTCTGAC-3’. The data are presented as the relative expression of target genes using the comparative threshold cycle (Ct) method (27).

### Cell Transfection

FTC-133R cells were transfected with non-targeting control scRNA (scRNA-cells) or siRNA specific to CD-47 (siRNA-cells) using Lipofectamine RNAiMAX following the Santa Cruz Biotechnology Inc (USA) the manufacturer’s instructions. The summary protocol: the cells were incubated in 6-well plates at 37°C in a CO_2_ incubator until they were 60–80% confluent, then washed with Transfection Medium. siRNA or scRNA transfection Reagent mixture (containing 50 pmol) was added and the cells were incubated 5–7 h at 37°C in a CO_2_ incubator. Cells were then washed and the efficiency of the CD47 mRNA depletion was checked out by RT-PCR after 24 h of culture period.

### Statistics

Each experiment was performed at least in triplicate with three independent sets of culture. Data were expressed as mean ± SEM. The statistical significance of differences was calculated using Student’s test. p values referring to corresponding control are NS, not significant; ** and ^××^ p < 0.01, *** and ^×××^ p < 0.001.

## Results

### Stepwise Selection of Multidrug Resistant-FTC-133 Cells Against Doxorubicin

**Figure 1:**
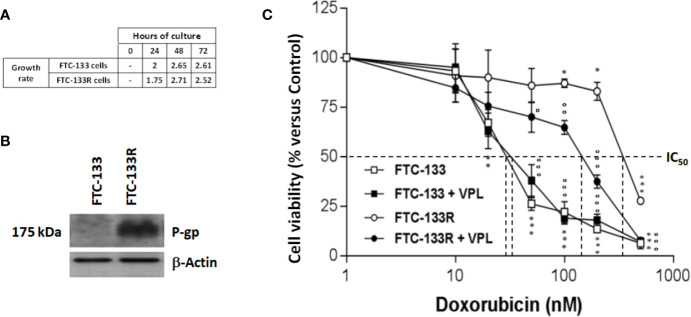
**(A)** FTC-133 and FTC-133R cells were cultured for 24, 48, and 72 h. Cell viability was measured using UptiBlue assay. Growth rate was calculated as ratio between D_n+1_/D_n_ **(B)**. FTC-133 and FTC-133R cells were cultured for 24 h. Detection of P-gp was evaluated by Western-blot. β-actin antibody was used as a control. A representative blot of three independent experiments was shown **(C)**. FTC-133 and FTC-133R cells were incubated with Dox at concentrations ranging from 10^-9^ to 10^-6^ M with or without 1 µM VPL. After 24 h, cell viability was measured using UptiBlue assay. Results were calculated as percent of control and represent mean ± standard deviation (S.D.) of at least three independent experiments. The statistical significance of differences was calculated using Student’s test. *p < 0.05, **p < 0.01, ***p < 0.001 compared to untreated cells. °p < 0.05, °°p < 0.01, °°°p < 0.001 compared to Dox-treated cells.

To establish a Dox-resistant FTC-133 cell line (FTC-133R cell), we progressively incubated FTC-133 cells with increasing concentrations of Dox ranging from 10 to 400 nM. FTC-133 cells which gradually adapted to the higher Dox concentration were named FTC-133 resistant cells (FTC-133-R). Cell observation by phase-contrast microscopy revealed no change in the morphology of FTC-133-R compared to the parental FTC-133 cells. In addition, no significant difference between the two cell lines growth rates was observed for a 24, 48, and 72 h period (**Figure 1A**).

### Implication of P-gp in FTC-133R Dox Resistance

**Figure 2:**
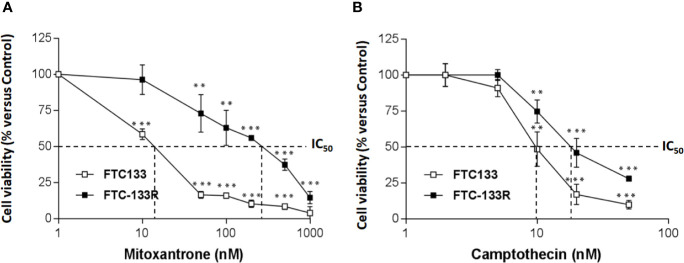
FTC-133 and FTC-133R cells were incubated with **(A)** Mitoxantrone at concentrations ranging from 10^-9^ to 10^-6^ M **(B)**, Camptothecin at concentrations ranging from 10^-9^ to 10^-7^ M. After 24 h, cell viability was measured using UptiBlue assay. Results were calculated as percent of control and represent mean ± standard deviation (S.D.) of at least three independent experiments. The statistical significance of differences was calculated using Student’s test. **p < 0.01, ***p < 0.001 compared to untreated cells.

To identify the mechanism of resistance to Dox, we first analyzed the expression of P-gp by western blot and we clearly showed a high level of P-gp expression in FTC-133R cells when compared to FTC-133 cells (**Figure 1B**). The chemo-sensitivity to Dox of resistant cells was compared to that of parental cells in the presence of increasing Dox concentrations for a period of 24 h (**Figure 1C**). As expected, Dox decreased FTC-133 cell viability in a dose-dependent manner with an IC_50_ = 30 nM. In contrast, FTC-133R cells exhibited a lower sensitivity to Dox, confirmed by a marked increase of IC_50_ to 580 nM. Thus, FTC-133R cells were 19-fold resistant to Dox than FTC-133 cells. Moreover, treatment of FTC-133R cells with VPL (1 µM), a standard P-gp inhibitor, induced a significant increase of Dox cytotoxicity. These results clearly established that P-gp blockade restored the FTC-133R cell sensitivity to Dox thus confirming that this process involved the P-gp transporter. In order to confirm the involvement of P-gp, cells were treated with mitoxantrone, another substrate of P-gp (28). IC_50_ of mitoxantrone was also higher in FTC-133R cells than in FTC133 cells (260 and 16 nM, respectively) (**Figure 2A**). Moreover, the analysis of camptothecin cytotoxicity, a topoisomerase I inhibitor which is not transported by P-gp, showed that IC_50_ of CPT was moderately higher in FTC-133R cells than in FTC133 cells (19 and 9.50 nM, respectively) (**Figure 2B**).

**Figure 3:**
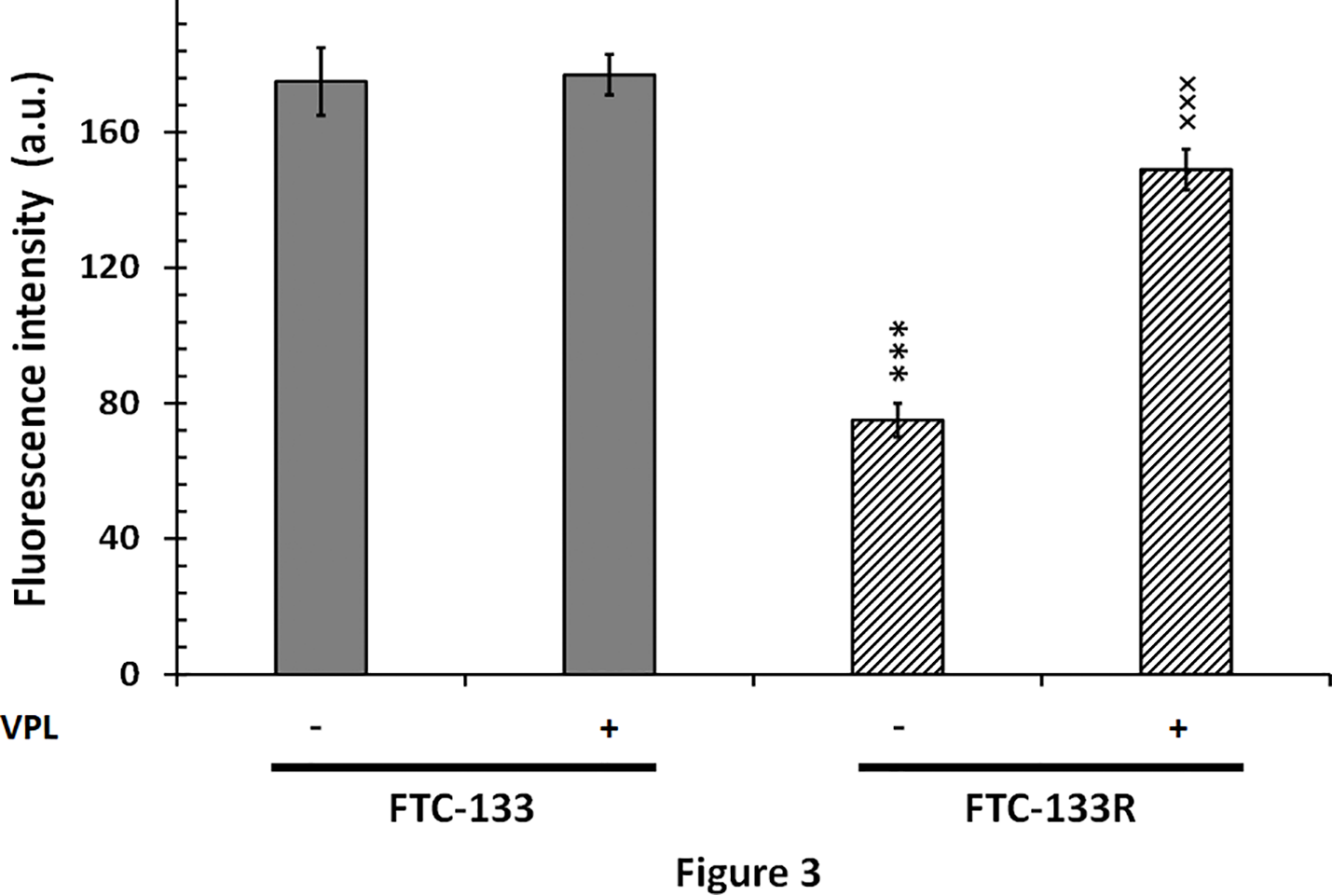
FTC-133 and FTC-133R cells were placed in petri dishes 24 h prior to the experiments and incubated with 4 µM Dox with or without 1 µM VPL for 5 h. The cells were washed with PBS free of drugs and placed in the medium without phenol red at 4˚C. The nuclear accumulation of Dox was monitored through its fluorescence emission spectra using confocal laser microspectrofluorometry. A nuclear spectrum of treated cells was obtained over the wavelength range 500–700 nm (26). The semi-quantification of nuclear Dox incorporation was obtained by measuring the fluorescence emission intensity of the band at 590 nm. Results represent mean ± standard deviation (S.D.) of at least three independent experiments. The statistical significance of differences was calculated using Student’s test. ***p < 0.001 versus FTC-133 and ^×××^p < 0.001 versus FTC-133R.

**Figure 4:**
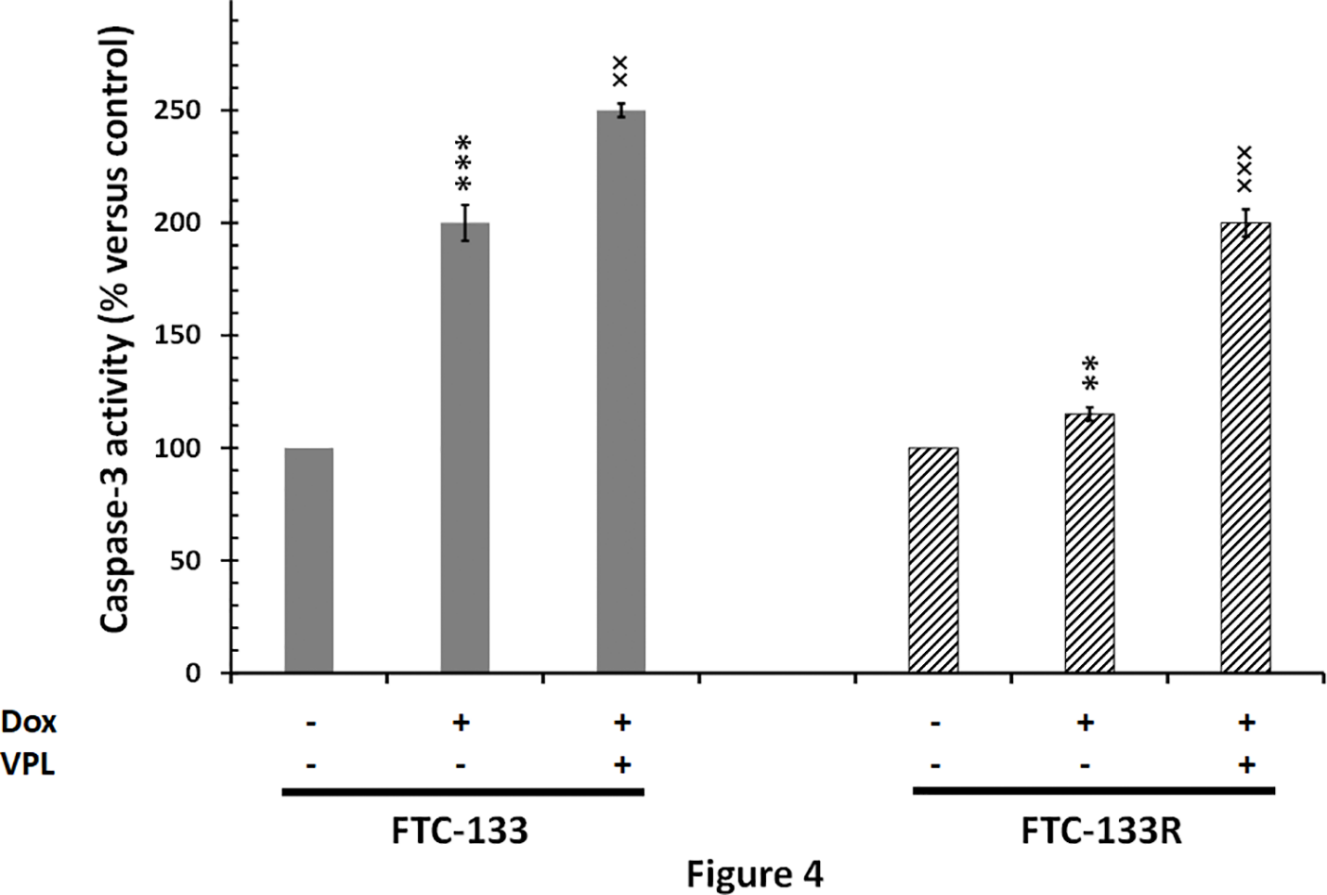
FTC-133 and FTC-133R cells were incubated with 0.1 μM Dox in the absence or presence of 1 µM VPL for 12 h. Caspase-3 activity was measured by caspACE assay kit. Results were calculated as percent of corresponding control and represent mean ± standard deviation (S.D.) of at least three independent experiments. The statistical significance of differences was calculated using Student’s test. **p < 0.01 and ***p < 0.001 versus control, ^××^p < 0.01 and ^×××^p < 0.001 versus Dox treatment.

Since cytotoxic activity of Dox depends on its intracellular localization, we then measured nuclear Dox fluorescence in both parental and resistant cells (**Figure 3**). We showed that nuclear Dox fluorescence was about 3 times weaker in FTC-133R cells than in FTC-133 cells. Addition of 1 µM VPL significantly increased drug nuclear accumulation in FTC-133R cells. This result confirmed the P-gp implication in FTC-113R chemoresistance to Dox by decreasing subcellular accumulation and cytotoxicity of Dox. To investigate whether resistance to Dox cytotoxicity in FTC-133R was associated to a protection against apoptosis, we analyzed the caspase-3 activity (**Figure 4**). We showed that Dox induced a very slight increase in the caspase-3 activity in FTC-133R cells when compared to FTC-133 cells. In presence of VPL, a significant increase in caspase-3 activity was observed in FTC-133R cells. This suggests that a lack in nuclear uptake of Dox in FTC-133R could be responsible for decrease of Dox cytotoxic effect and low level of caspase-3 activation.

### Correlation Between P-gp and CD4/TSP-1 Expression in FTC-133 Resistant Cells

**Figure 5:**
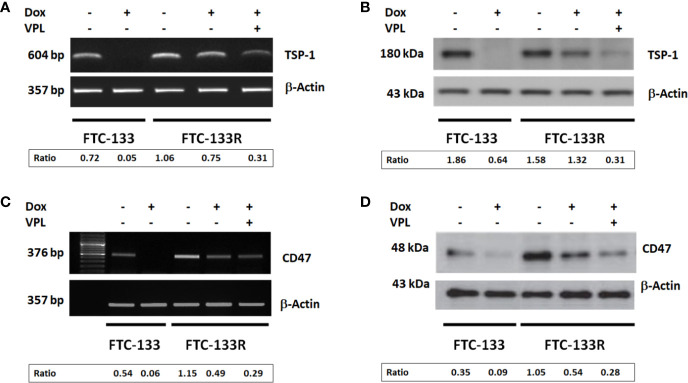
FTC-133 and FTC-133R cells were incubated with 0.1 μM Dox in the absence or presence of 1 µM VPL for 12 h **(A)**. TSP-1 mRNA expression was evaluated by RT–PCR. The constitutively expressed β-actin gene was used as a normalizing control **(B)**. TSP-1 protein secreted in the culture medium was detected by Western blot. β-actin antibody was used as a control **(C)**. CD47 mRNA expression was evaluated by RT–PCR. The constitutively expressed β-actin gene was used as a normalizing control **(D)**. CD47 protein was detected by Western blot. A representative agarose gel electrophoresis and blot of three independent experiments was shown. The intensity of the bands was quantified by densitometry using quantity one program.

The microenvironment has emerged as a key player in the development of chemoresistance (16, 17, 29, 30). In fact, we previously reported that TSP-1/CD47 interaction could play a role in cell resistance to Dox (23, 24). So, we analyzed the expression of TSP-1 and CD47 in FTC-133R cells. PCR and western blot analysis showed that FTC-133R cells expressed similar levels of TSP-1 mRNA and protein as compared to FTC-133 cells (**Figures 5A, B**). Nevertheless, TSP-1 expression was dramatically abolished by Dox at both mRNA and protein levels in FTC-133 cells while slightly reduced in FTC-133R cells. This effect was amplified by VPL, thus suggesting that P-gp could contribute to maintain TSP-1 expression level. We then analyzed CD47 expression and we showed that CD47 mRNA and protein levels were markedly increased in FTC-133R cells as compared to FTC-133 cells (**Figures 5C, D**). In addition, following Dox treatment, CD47 mRNA and protein expression were dramatically decreased in FTC-133 cells while slightly reduced in FTC-133R cells. This effect was however amplified when Dox treatment was associated to VPL. These results suggest that CD47 overexpression in FTC-133R cells may contribute to cell protection against chemotherapy.

**Figure 6:**
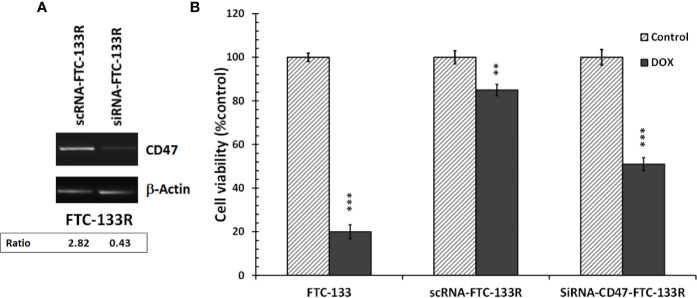
FTC-133R cells were transfected with nontargeting control scRNA (scRNA-FTC-133R) or siRNA specific to CD47 (siRNA-CD47-FTC-133R) using Lipofectamine RNAiMAX **(A)**. CD47 mRNA levels were analyzed by RT-PCR on total RNA prepared by RNeasy^®^ Mini kit. β-actin mRNA was used as a control. A representative agarose gel electrophoresis of three independent experiments was shown **(B)**. FTC-133, scRNA-FTC-133R and siRNA-CD47-FTC-133R cells were incubated with 0.1 μM Dox for 24 h. Cell viability was measured using UptiBlue assay. Results were calculated as percent of corresponding control and represent mean ± standard deviation (S.D.) of at least three independent experiments (**p < 0.01 and ***p < 0.001 versus control). The intensity of the bands was quantified by densitometry using quantity one program.

**Figure 7:**
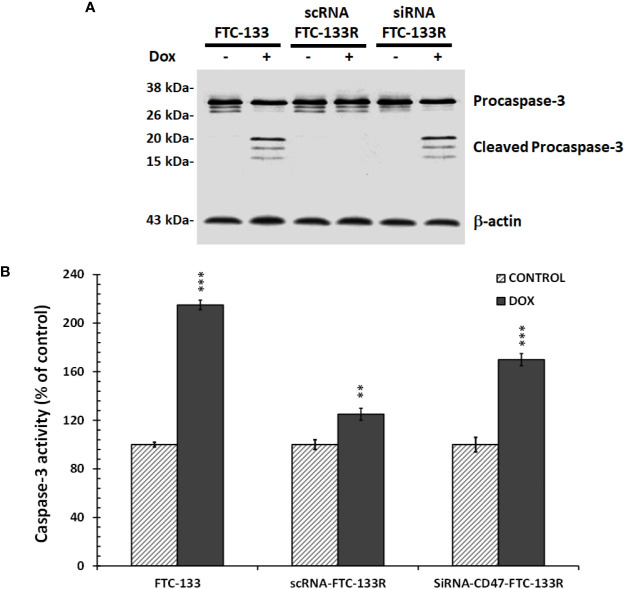
FTC-133, scRNA-FTC-133R, and siRNA-CD47-FTC-133R cells were incubated with 0.1 μM Dox for 12 h **(A)**. Procaspase-3 and cleaved caspase-3 were detected by Western blotting. β-actin antibody was used as a control. A representative blot of three independent experiments was shown **(B)**. Caspase-3 activity was measured by caspACE assay kit. Results were calculated as percent of corresponding control and represent mean ± standard deviation (S.D.) of at least three independent experiments (**p < 0.01 and ***p < 0.001 versus control).. The intensity of the bands was quantified by densitometry using quantity one program.

To confirm the role of CD47, its expression was silenced by RNA interference (siRNA) in FTC-133R cells. The efficiency of the transfection was controlled by RT-PCR and showed that CD47 expression was decreased by 83% in siRNA-CD47 transfected cells (siRNA-CD47-FTC-133R) whereas it remained unchanged in scrambled siRNA transfected cells (scRNA-FTC-133R) (**Figure 6A**). Cell viability analysis showed that silencing of CD47 mRNA sensitized FTC-133R to Dox treatment by decreasing cell viability (**Figure 6B**). Indeed, The IC_50_ of Dox was decreased from 450 nM (in scRNA-FTC-133R cells) to 320 nM (in siRNA-CD47-FTC-133R cells) (data not shown). Then, we determined whether these data could be correlated to apoptosis process by analysing caspase-3 activation. Western blot analysis showed that Dox induced an increase in pro-caspase-3 cleavage in FTC-133 cells but had no effect in scRNA-FTC-133R cells (**Figure 7A**). These results were correlated to data obtained on caspase-3 activity (**Figure 7B**). Silencing of CD47 in FTC-133R (siRNA-CD47-FTC-133R) cells restored the pro-apoptotic role of Dox through caspase-3 activation. These results confirmed the contribution of CD47 overexpression in FTC-133R cell to protection against Dox-induced apoptosis.

### Anti-Apoptotic Effect of TSP-1/CD47 Interaction in FTC-133R Cells Through Blocking JNK Phosphorylation

**Figure 8:**
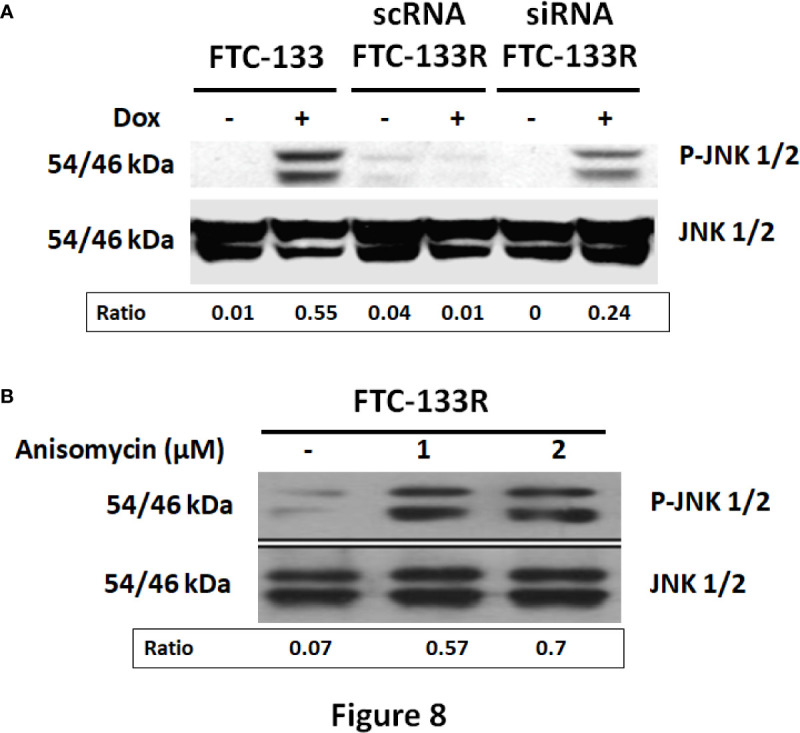
**(A)** FTC-133, scRNA-FTC-133R, and siRNA-CD47-FTC-133R cells were incubated for 12 h with or without 0.1 μM Dox. Detection of p-JNK and JNK were evaluated by Western-blot **(B)**. FTC-133R cells were incubated with anisomycin (1 and 2 µM) for 12 h. Detection of p-JNK and JNK were evaluated by Western-blot. A representative blot of three independent experiments was shown. The intensity of the bands was quantified by densitometry using quantity one program.

**Figure 9:**
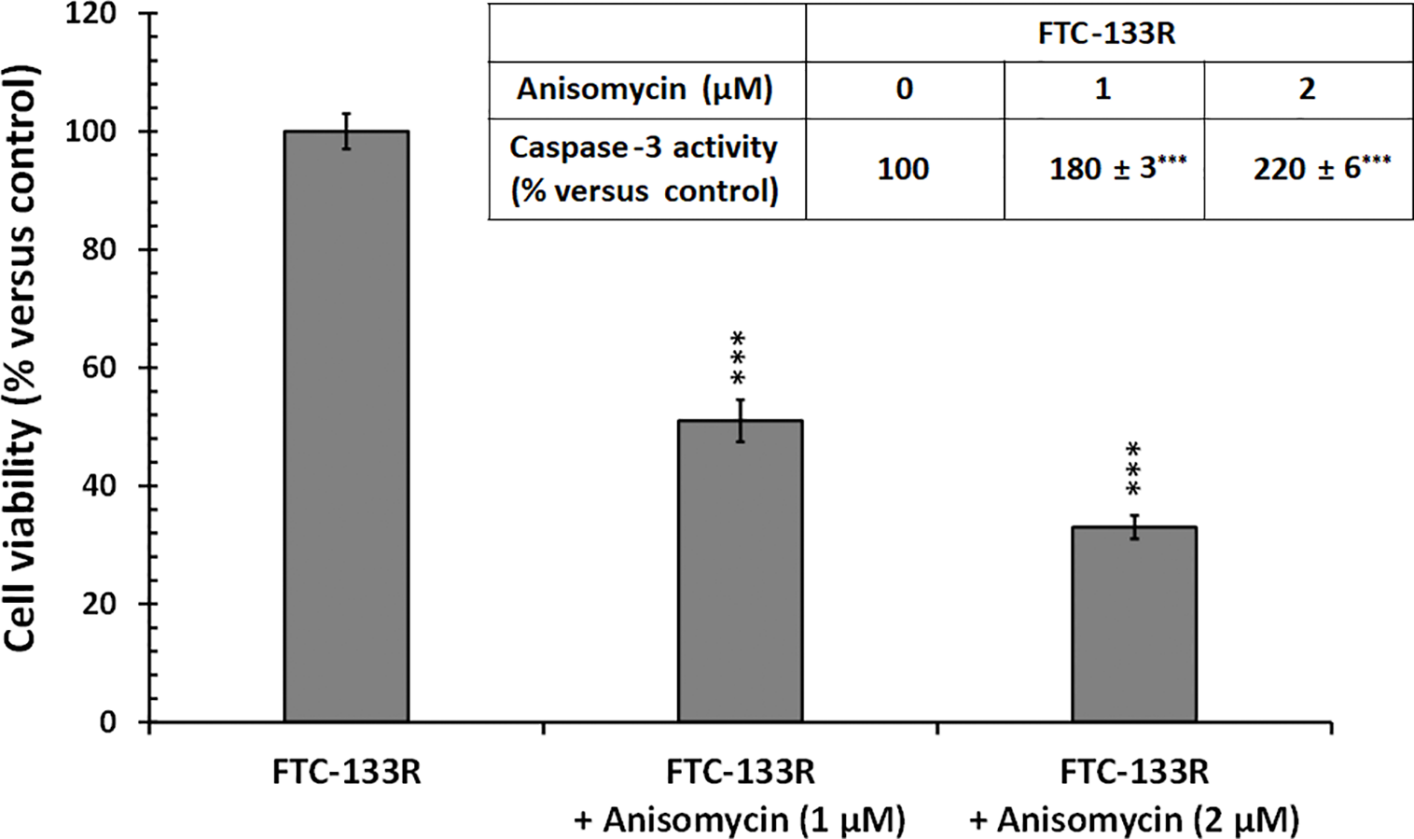
FTC-133R cells were incubated for 24 h with or without anisomycin (1 and 2 µM), cell viability was measured using UptiBlue assay. Insert: FTC-133R cells were incubated for 12 h with or without anisomycin (1 and 2 µM). Caspase-3 activity was measured by caspACE assay kit. Results were calculated as percent of corresponding control and represent mean ± standard deviation (S.D.) of at least three independent experiments (***p < 0.001 versus control).

In our previous studies, we have shown that JNK signalling pathway was involved in Dox-induced apoptosis of FTC-133 cells (23). Thus, we analyzed JNK phosphorylation in FTC-133 and FTC-133R cells (**Figure 8**). Western blot analysis showed that Dox induced JNK phosphorylation in FTC-133 cells but had not effect in FTC-133R cells (**Figure 8A**). However, Dox was able to induce JNK phosphorylation in siRNA-CD47-FTC-133R. These results suggested the implication of TSP-1/CD47 interaction in the protection against apoptosis in FTC-133R cells through blocking JNK phosphorylation. To confirm the involvement of JNK, we used the JNK agonist, anisomycin. As expected, anisomycin (1 and 2 µM) induced JNK phosphorylation in FTC-133R cells in a dose-dependent manner (**Figure 8B**). We then investigated whether JNK phosphorylation could affect FTC-133R cell resistance to the cytotoxic and apoptotic effects of Dox. As shown in **Figure 9**, anisomycin decreased FTC-133R cell viability in a dose-dependent manner and induced an increase in capsase-3 activity. These data suggest that activation of JNK pathway allows to circumvent resistance of FTC-133R cells to the cytotoxic and apoptotic effects of Dox.

## Discussion

Several cellular phenomena contribute to MDR, including overexpression of drug efflux pumps, induction of cell survival pathways, and resistance to apoptosis. Drug efflux is based on the overexpression of ABC transport proteins, such as P-glycoprotein (P-gp), multidrug resistance-associated protein (MRP) and breast cancer resistance protein (BCRP) (1, 4, 5). Apart from cellular events, microenvironmental factors such as remodeling of the extracellular matrix (ECM) are implicated in MDR (31, 32). Since the last few years, several studies have shown that the tumor microenvironment modulates cell response to chemotherapy and it is clearly now admitted that this ECM-mediated effect contributes to a new form of *de novo* resistance (14, 33–35). The Dalton’s group was the first to show that fibronectin was able to protect tumor cells from drug-induced apoptosis *via* the β-integrin/PI3-kinase signaling (14, 16, 17, 29, 30, 33, 36). This protective effect has also been described for vitronectin, another ECM protein (37). Conversely, some tumor stroma components are able to sensitize cancer cells to anti-cancer drugs. This is the case with the reduction of TGF in ovarian carcinoma cells treated with paclitaxel and which could be a poor prognosis for patients (19, 38).

Thrombospondin-1 (TSP-1), another ECM component composed of multiple domains, interacts with various partners leading to different effects. Indeed, TSP-1 induces apoptosis of endothelial cells *via* the CD36 receptor but also modulates tumor cell response to chemotherapy *via* the CD47 receptor (20, 39, 40).

We previously reported that Dox-induced apoptosis of human thyroid carcinoma FTC-133 cells *via* JNK/ATF-2 activation. Moreover, this effect was accompanied by a down regulation of TSP-1 expression. Addition of exogenous TSP-1 or its derived peptide 4N1 protects FTC-133 cells against Dox-induced apoptosis. This effect is mediated by TSP-1 C-terminal domain interaction with the membrane receptor CD47 in FTC-133 cells (21–23). These findings suggest that induction of apoptosis by Dox in FTC-133 cells is greatly dependent on a down-regulation of TSP-1 expression and shed new light on a possible role for TSP-1 in drug resistance. However, a link between tumor microenvironment and ABC transporter­related MDR remains a matter of debate.

In this study, we investigated how TSP-1 contributed to FTC-133 cell *novo* resistance to Dox-induced apoptosis and thereby affected the development of acquired drug resistance. To that end, we have selected resistant cells (FTC-133R) from the parental FTC-133 cells which overexpressed the ABC transporter P-gp and were 19-fold resistant to Dox as shown by the drug cytotoxic effect and measurement of nuclear drug uptake. Moreover, the P-gp antagonist VPL restored Dox cytotoxicity in FTC-133R cells confirming that P-gp is the predominant mechanism of acquired resistance. Indeed, it has been shown that the overexpression of P-gp in cancer was either an inherent or acquired process: the former, a reflection of its physiologic expression, and the latter, generated by the presence of anticancer drugs (41). P-gp confers resistance by preventing sufficient accumulation of anticancer drugs within the cell, thereby avoiding their cytotoxic or apoptotic effects (41).

This resistance was accompanied by a CD47 elevated expression whereas TSP-1 expression was not affected. Other studies confirmed the overexpression of integrins such as α4β1 and α5β1 in Dox-resistant 8226 myeloma cells and that integrin-mediated adhesion induced apoptosis resistance (42). In the same context, acquisition of MDR in MCF7 cells was associated with markedly decreased expression of α2β1 and αvβ3 integrin’s and dramatic up-regulation of α5β1 integrin. Stimulation of β1 integrin signaling strongly sensitizes MCF-7 cells to anoikis (43). In our study, CD47 and TSP-1 expression were correlated to P-pg activity. In fact, inhibition of P-gp activity in FTC-133R cell by VPL significantly reduced both CD47 and TSP-1 expression and sensitized cells to Dox. These results corroborated our previous data reporting that TSP-1/CD47 interaction protected against Dox-induced apoptosis in FTC-133 cells (22, 23). Our studies also suggested that P-gp-overexpressed cells were able to promote the anti-apoptotic role conferred by ECM components. Moreover, prevention of TSP-1/CD47 interaction by siRNA specific to CD47 abolished the TSP-1 anti-apoptotic effect and reduced the resistance to Dox in FTC-133R. Our results clearly showed that CD47 expression was able to regulate cell resistance to Dox despite the P-gp overexpression and confirmed that the microenvironment may contribute and/or potentiate the acquired Dox resistance. Several studies showed that the tumor microenvironment disabled cytotoxic effect of some chemotherapeutic agents resulting in resistance and failure in drug response either through disturbing drug partitioning, sequestering it intracellularly (31, 44), or through induction of P-gp expression (45, 46). Tatsuta et al (47). showed that ECM components modulated the P-gp expression in brain capillary endothelial cells (47). In the same perspective, Naci et al (18). confirmed that collagen/β1 integrin interaction increased MRP-1 expression in leukemic T-cells, which consequently decreased the amount of intracellular Dox and Dox-induced apoptosis (18).

Our previous data reported that the JNK/ATF-2 signaling pathway was involved in Dox-induced apoptosis in FTC-133 cells (22). Here, we showed that JNK was weakly phosphorylated following Dox treatment in FTC-133R cells. In addition, TSP-1/CD47 interaction contributed to decrease JNK activation and thus protected resistant cells from Dox-induced apoptosis. Moreover, pharmacological JNK activation bypassed resistance and restored the drug cytotoxicity in resistant cell that overexpressed P-pg. Thus, JNK signaling pathway maybe further considered as a relevant target for a novel approach to overcome chemoresistance in thyroid carcinoma. This is in agreement with other that have also reported the impact of JNK on cancer progression and therapy (48, 49).

In conclusion, our study shows that tumor cell microenvironment can modulate the response of cancer cell to chemotherapeutic treatment. Our study demonstrates an important survival role for CD47 and its ligand TSP-1 in Dox-induced apoptosis of FTC-133 cell and thus contribute to the modulation of P-gp drug resistance. Molecular characterization of acquired resistance must take into consideration the interaction of tumor cells with their microenvironment in order to identify new targets of drug resistance.

## Data Availability Statement

The original contributions presented in the study are included in the article/supplementary material. Further inquiries can be directed to the corresponding author.

## Author Contributions

M-PC, HM, LD, HE: performing biochemistry, cell, and molecular biology experiments. LM, ED-C, HM, HE: analysis and interpretation of data. ED-C, HM, HE: drafting of manuscript. M-PC, LM: critical revision. All authors contributed to the article and approved the submitted version.

## Funding

This work was supported by the University of Reims Champagne Ardennes and the Centre National de la Recherche Scientifique (CNRS).

## Conflict of Interest

The authors declare that the research was conducted in the absence of any commercial or financial relationships that could be construed as a potential conflict of interest.
